# Supplementary material for: Gene-Delivery Ability of New Hydrogenated and Partially Fluorinated Gemini bispyridinium Surfactants with Six Methylene Spacers
Source: Int J Mol Sci. 2022 Mar 11;23(6):3062. doi: 10.3390/ijms23063062 (PMC8949414; doi:10.3390/ijms23063062)
Supplement: Supplementary file 1 [file ijms-23-03062-s001.zip › ijms-1602242-supplementary.pdf]

SUPPORTING INFORMATION

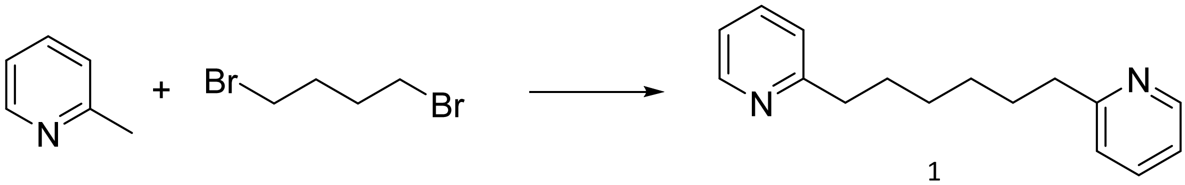

Reagents and conditions: Diethyl ether, BuLi, -20 °C

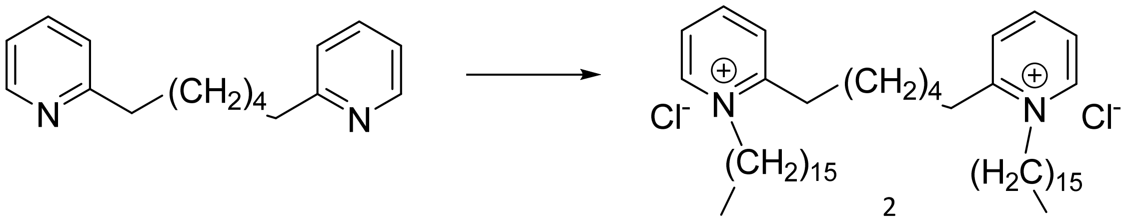

Reagents and conditions: DMF, hexadecyl chloride, 150 °C

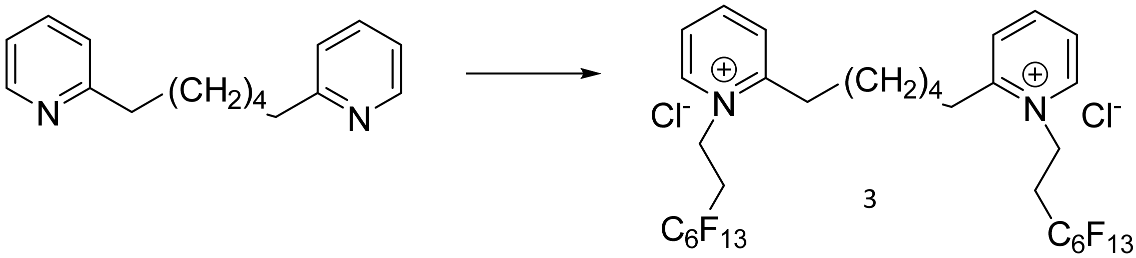

Scheme S1. Reagents and conditions: DCM, 1,1,1,2,2,3,3,4,4,5,5,6,6-tridecafluoro-8-(methylsulfonyl) octane chloride, 60 °C

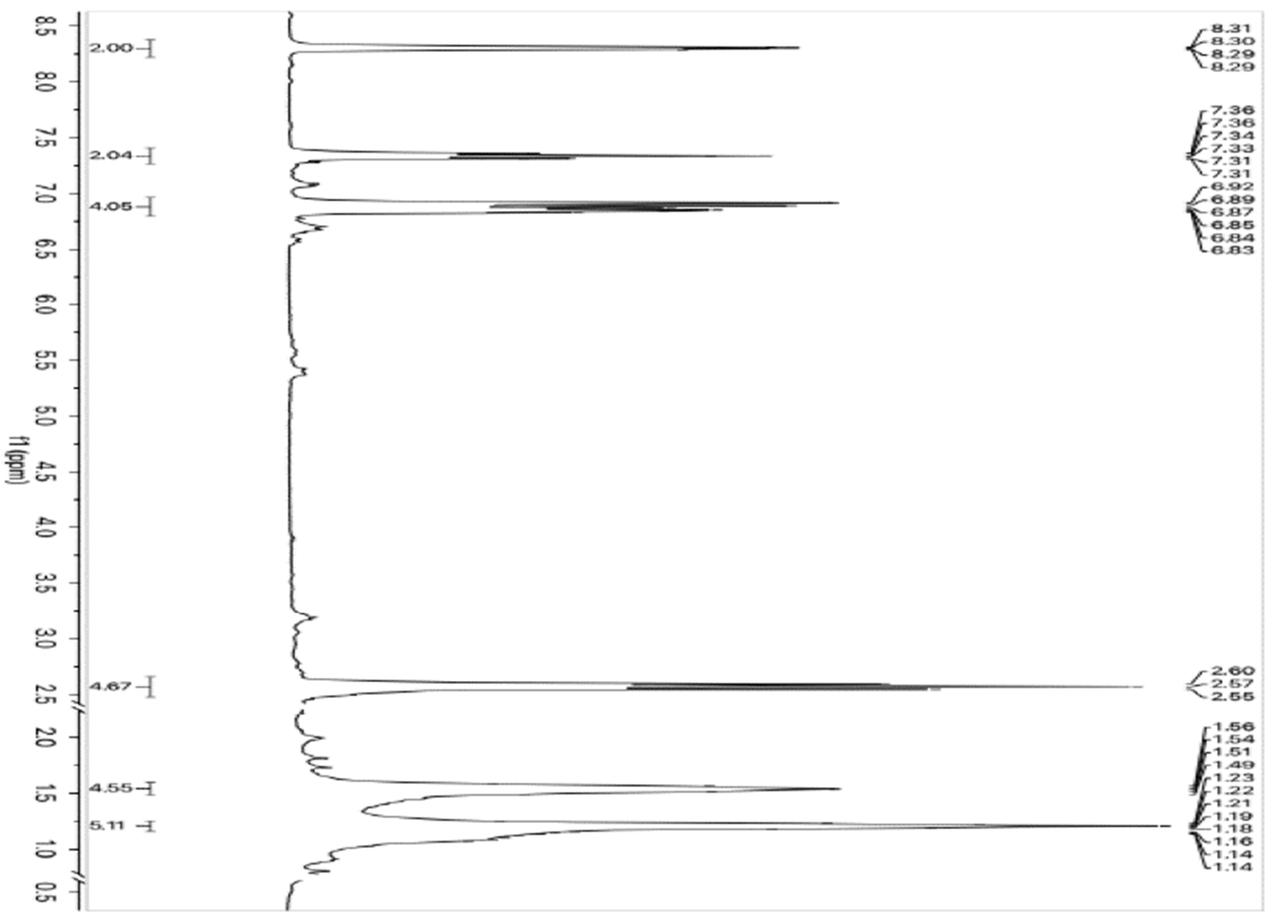

Figure S1. ^{1}H-NMR of 2-[6-(pyridine-2-yl)hexyl]pyridine.

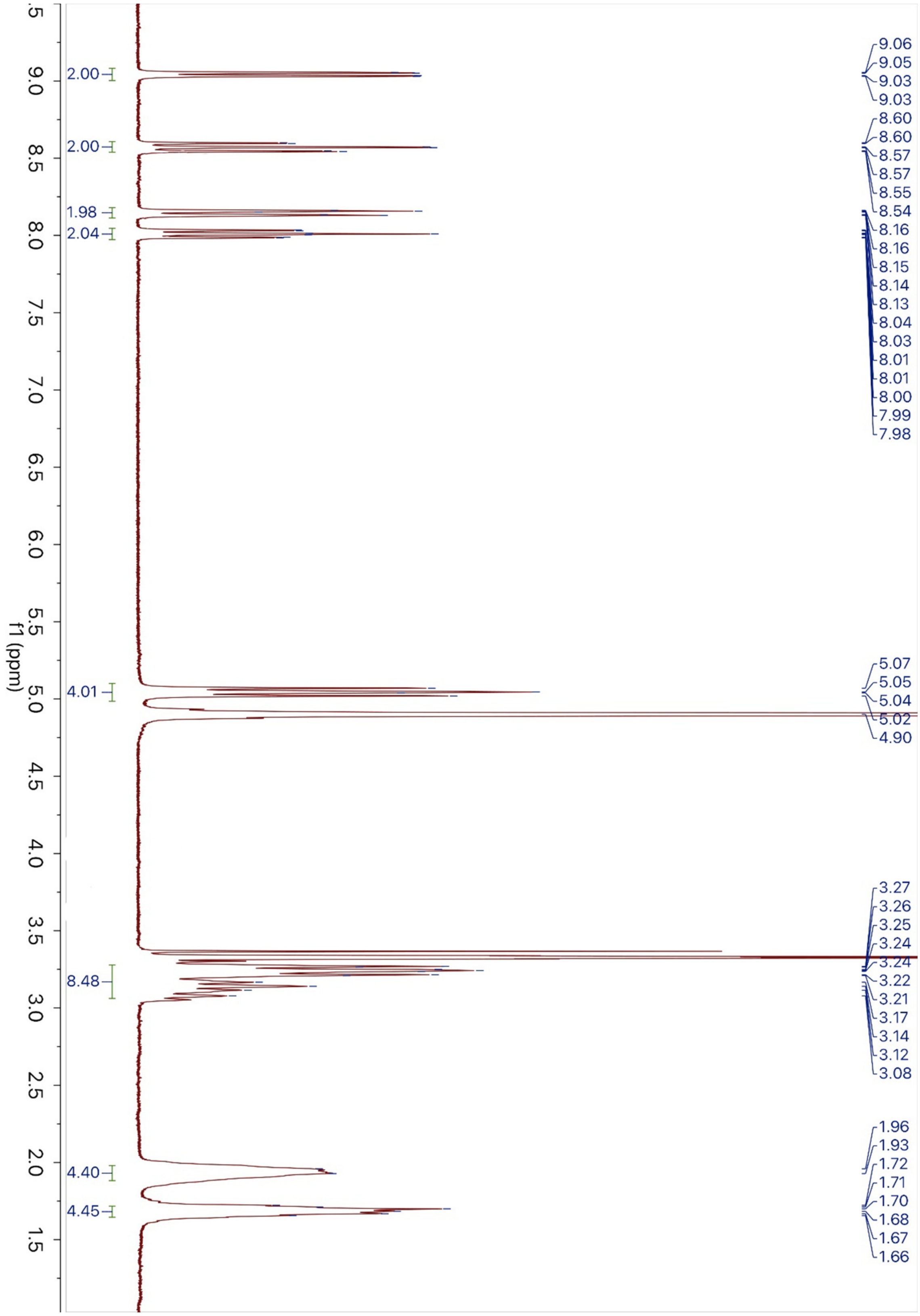

Figure S2. ^{1}H-NMR of 1,1’-bis(3,3,4,4,5,5,6,6,7,7,8,8-tridecafluorooctyl)-2,2’-esamethylenebispyridinium chloride (FGP6).

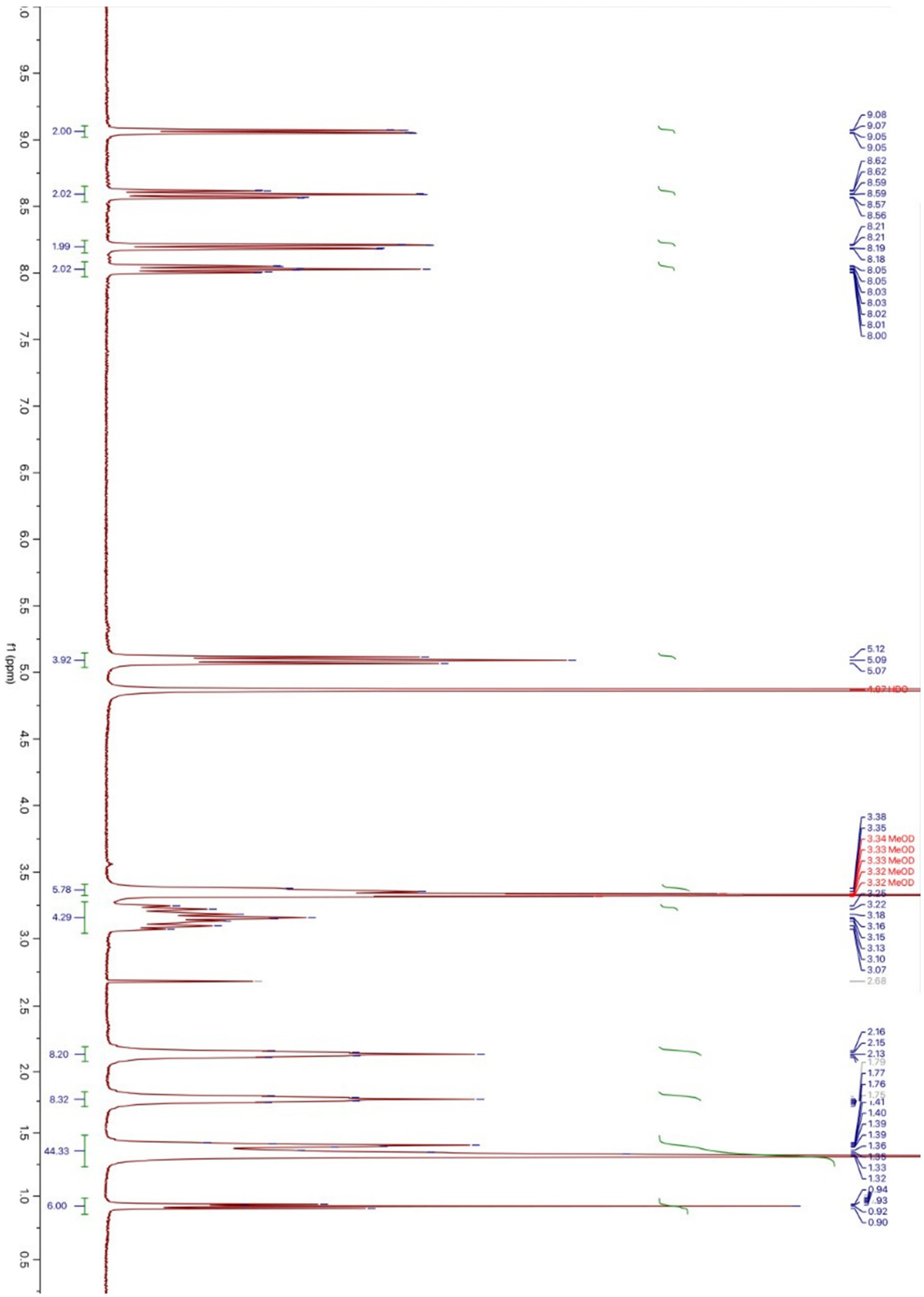

Figure S3. ^{1}H-NMR of 1,1'-bis-hexadecyl-2,2'-hexamethylenebispyridinium chloride (GP16_6).

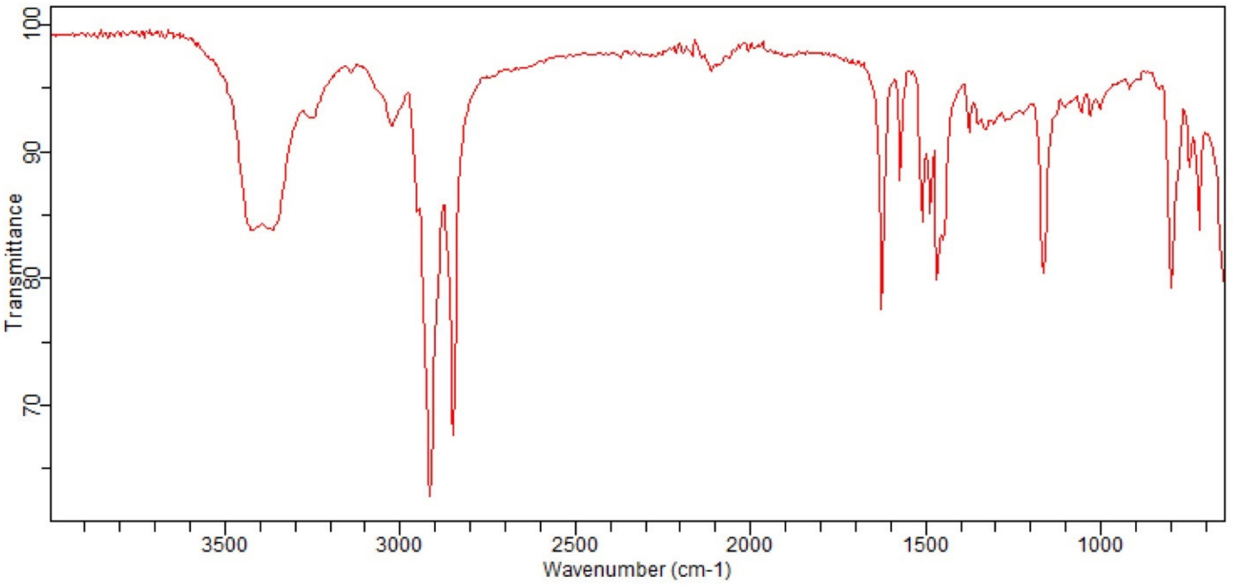

Figure S4. IR spectrum of 1,1’-bis-hexadecyl-2,2’-hexamethylenebispyridinium chloride (GP16_6).

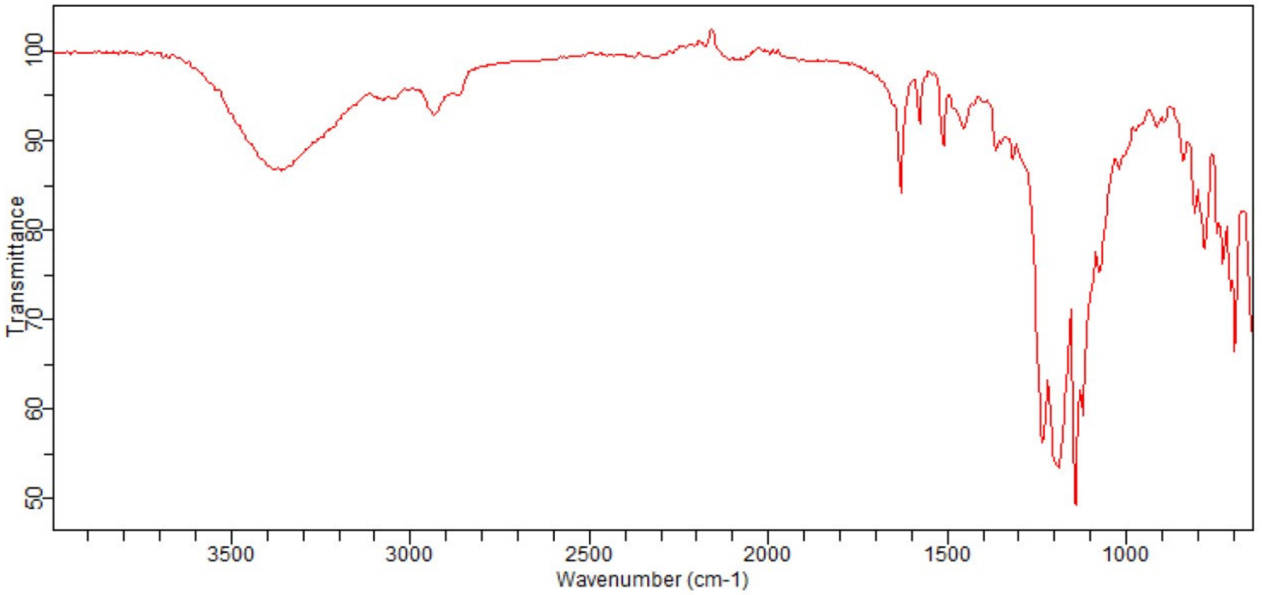

Figure S5. IR spectrum of 1,1’-bis(3,3,4,4,5,5,6,6,7,7,8,8-tridecafluorooctyl)-2,2’-esamethylenebispyridinium chloride (FGP6).

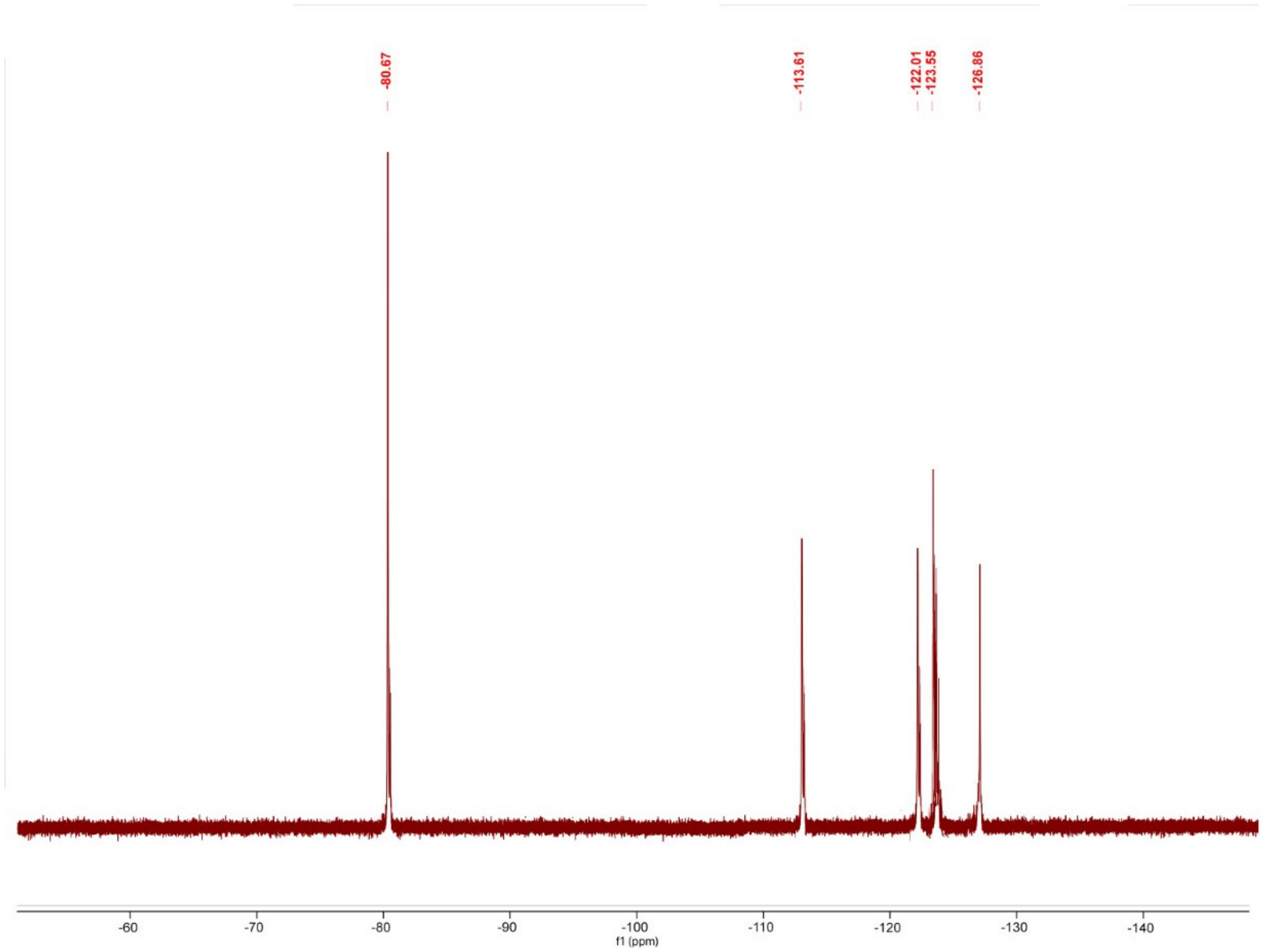

Figure S6. ^{19}F-NMR of 1,1’-bis(3,3,4,4,5,5,6,6,7,7,8,8-tridecafluorooctyl)-2,2’-esamethylenebispyridinium chloride (FGP6).

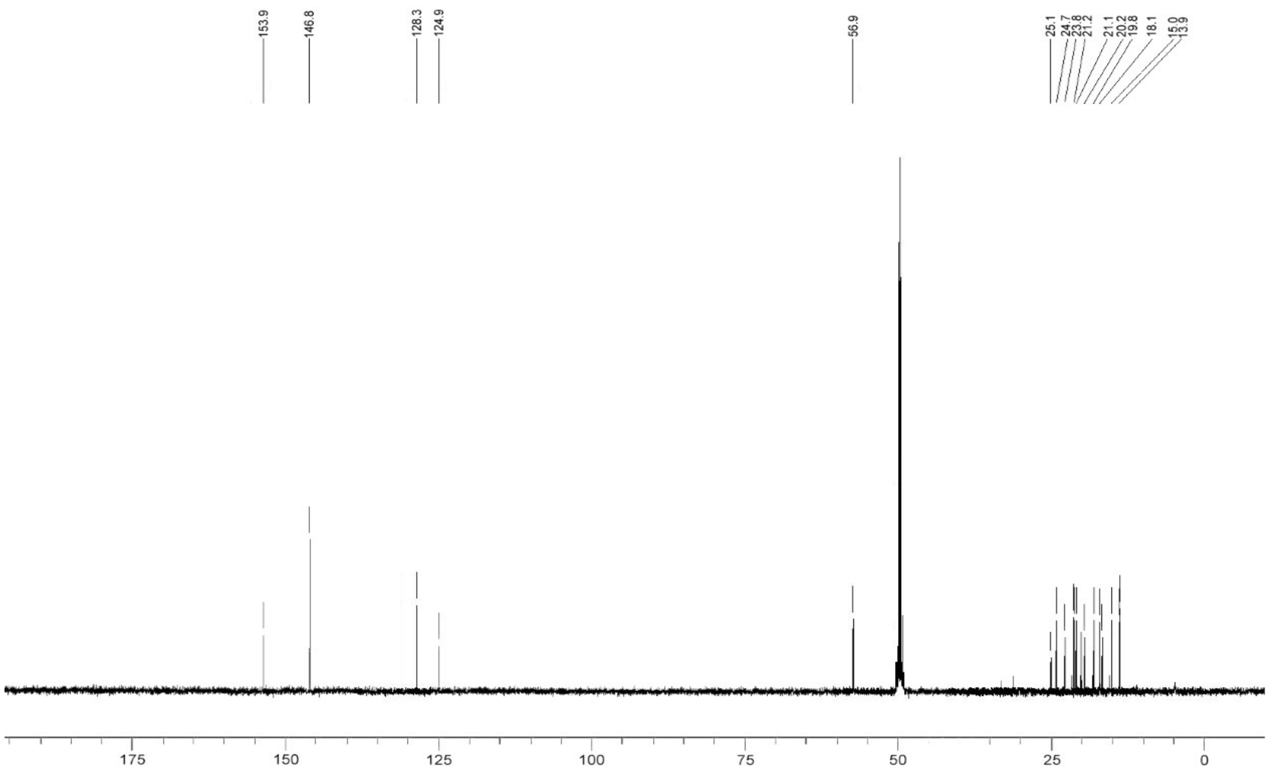

Figure S7. ^{13}C-NMR of 1,1'-bis-hexadecyl-2,2'-hexamethylenebispyridinium chloride (GP16_6).

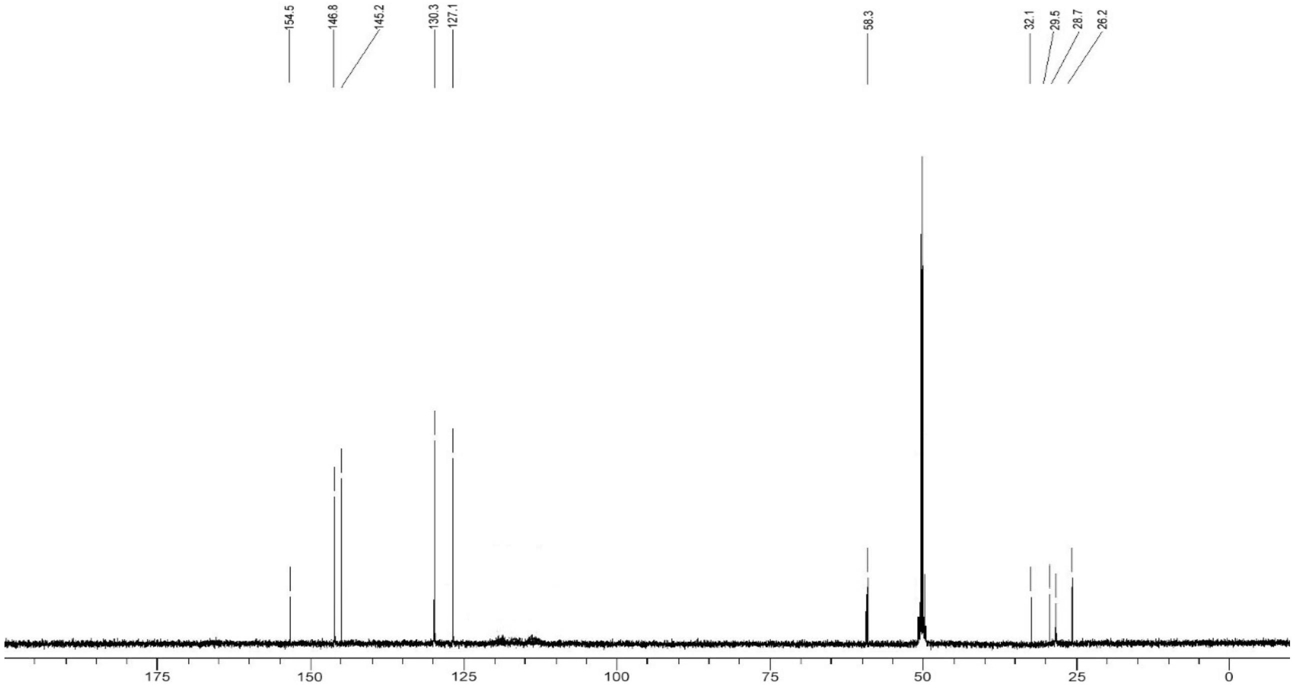

Figure S8. ^{13}C-NMR of 1,1'-bis(3,3,4,4,5,5,6,6,7,7,8,8-tridecafluorooctyl)-2,2'-esamethylenebispyridinium chloride (FGP6).

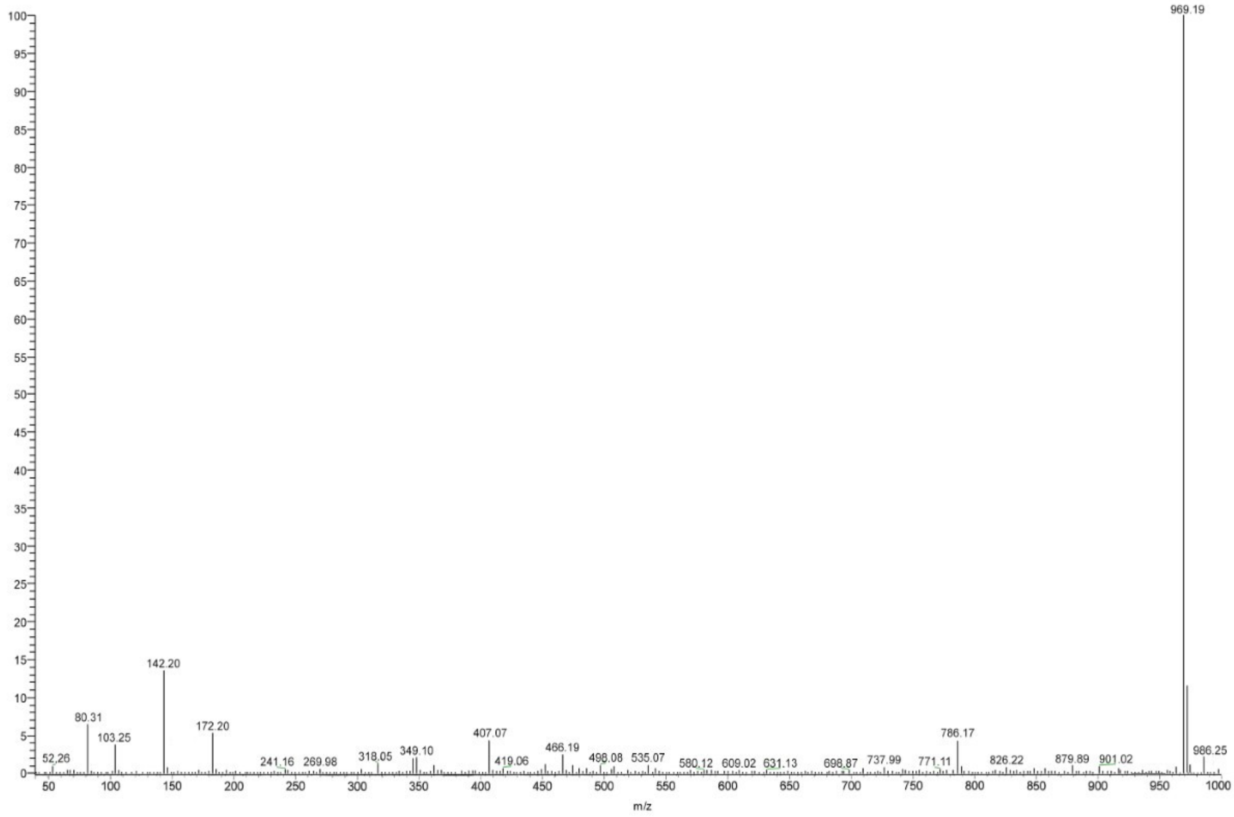

Figure S9. Mass spectrum of 1,1'-bis(3,3,4,4,5,5,6,6,7,7,8,8-tridecafluorooctyl)-2,2'-esamethylenebispyridinium chloride (FGP6).

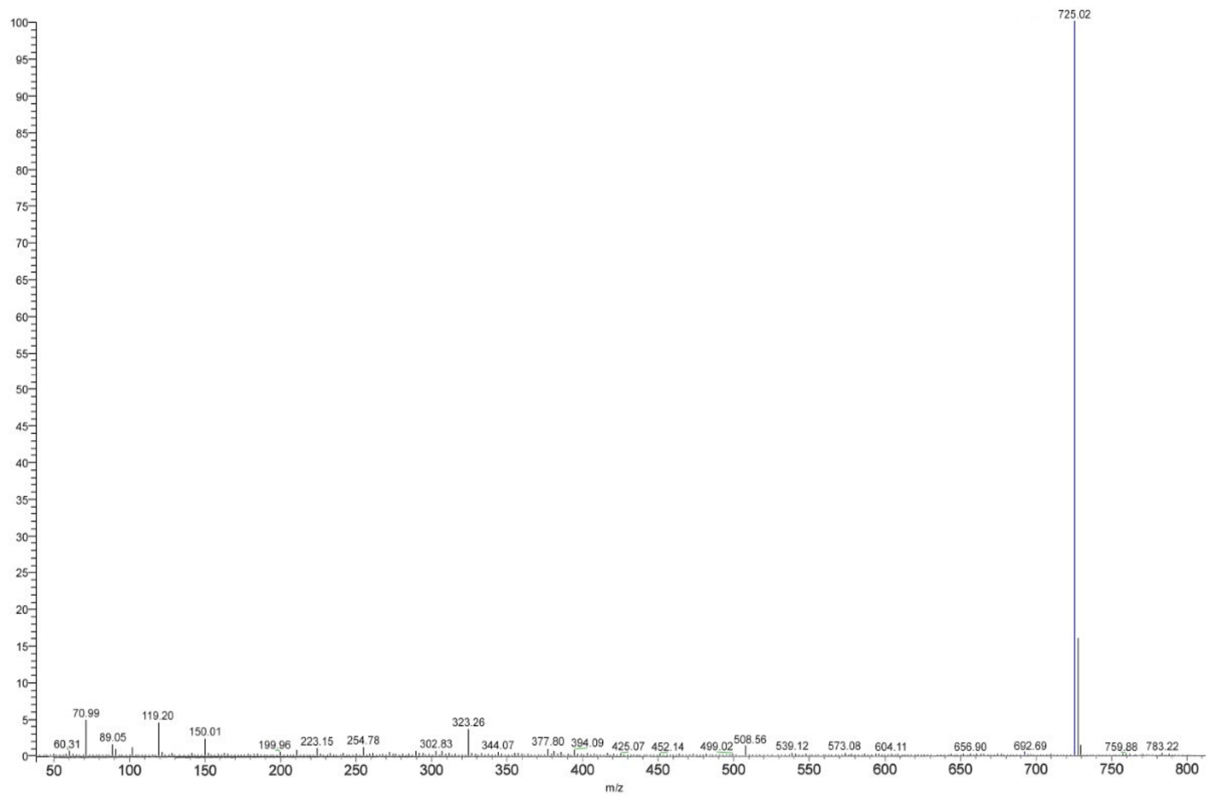

Figure S10. Mass spectrum of 1,1'-bis-hexadecyl-2,2'-hexamethylenebispyridinium chloride (GP16_6).

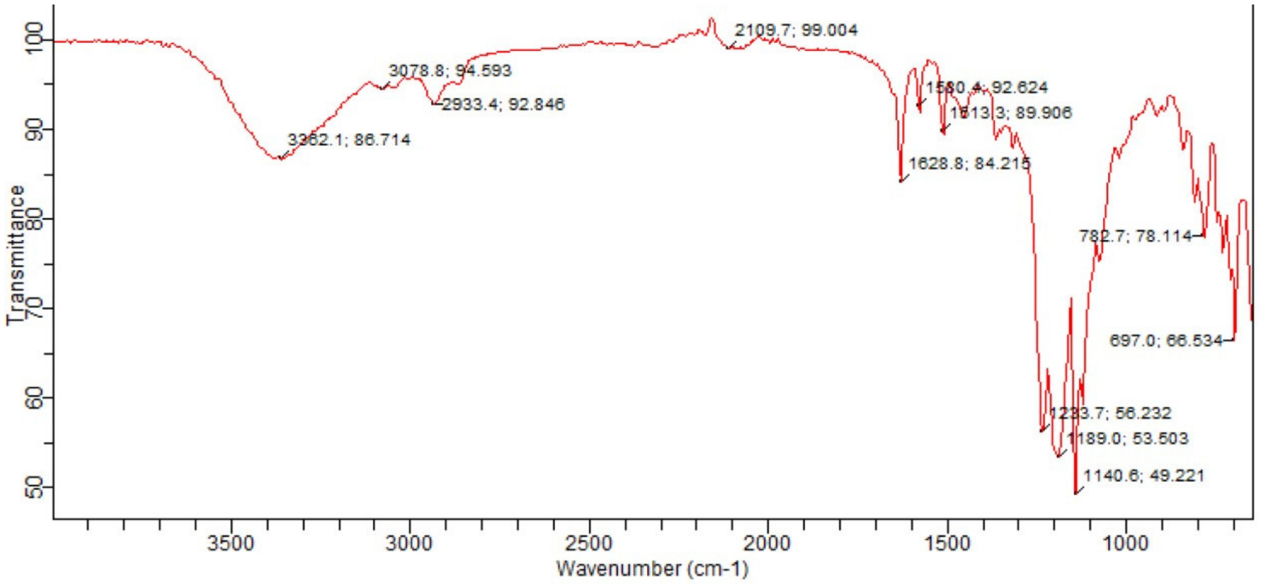

Figure S11. IR spectrum of 1,1’-bis(3,3,4,4,5,5,6,6,7,7,8,8-tridecafluorooctyl)-2,2’-esamethylenebispyridinium chloride (FGP6).

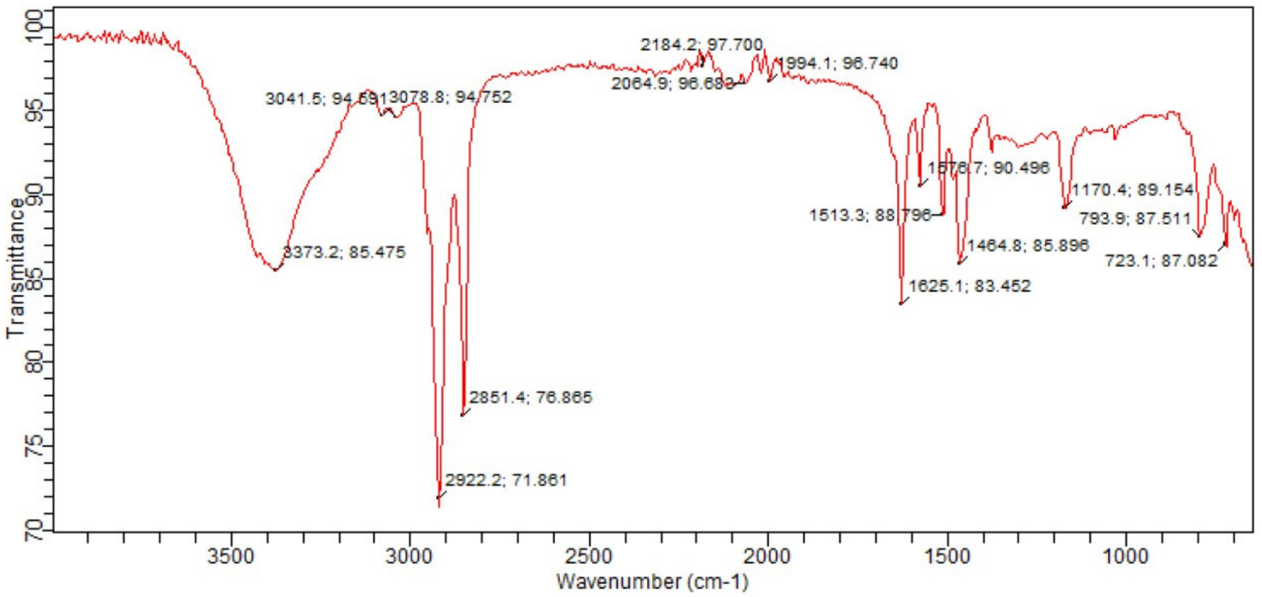

Figure S12. IR spectrum of 1,1’-bis-hexadecyl-2,2’-hexamethylenebispyridinium chloride (GP16_6).
